# Arterio venous fistula experience at a tertiary care hospital in Pakistan

**DOI:** 10.12669/pjms.291.2753

**Published:** 2013

**Authors:** Shah nawaz, Shahzad Ali, Iqbal Shahzad, M.Umar Baloch

**Affiliations:** 1 Dr. Shahnawaz, MS, Department of Urology, Jinnah Postgraduate Medical Centre, Karachi, Pakistan.; 2 Dr. Shahzad Ali, FCPS, Department of Urology, Jinnah Postgraduate Medical Centre, Karachi, Pakistan.; 3 Dr. Iqbal Shahzad, FCPS, Department of Urology, Jinnah Postgraduate Medical Centre, Karachi, Pakistan.; 4 Dr. Muhammad Umar Baloch, MS, Department of Urology, Jinnah Postgraduate Medical Centre, Karachi, Pakistan.

**Keywords:** Arteriovenous fistula, AVF, Primary failure, Patency, Chronic renal failure

## Abstract

***Objective: ***To evaluate the two year patency rate of functioning arteriovenous fistula.

***Methodology: ***This prospective case series study was conducted at Department of Urology, Jinnah Postgraduate Medical Centre, Karachi, from 1^st^ January 2009 to 31^st^ December, 2010. Patients were chosen for CBRC arteriovenous fistula at wrist and patients undergoing other types of vascular access or secondary fistula formation were excluded.

***Results:***One hundred and eighty two patients underwent arteriovenous fistula formation. The mean ± SD age was 63 ± 13 years and there were 102 (56%) males and 80 (44%) females. 12.6% fistulae failed within first month without dialysis. The primary patency rate was 66.5% at three months and 57.7% at six months. Failing arteriovenous fistula was managed by new arteriovenous fistula in our series. 28.6% patients had redo arteriovenous fistula. This study demonstrated a poor outcome for fistulas in diabetic patients. Fifteen out of 23 (65.2%) who failed primarily were diabetics and out of these diabetics 13 (86.7%) failed in first three months. Infection and burst fistulae were found in nine (4.9%), pseudo aneurysm in 3.2%, fever 4.9%, peri-operative failure 0.55% and burst fistulae 3.2%.

***Conclusions:*** One-third of radiocephalic fistulas fail within two years. The outcome is worse for women and diabetic patients. This information may be useful in assessing and counseling patients with end-stage renal failure. Arteriovenous fistula is the better and ideal choice for haemodialysis. A Radiocephalic fistula in forearm seems to have better results as comparison to cubital fossa arteriovenous fistula. End to side anastomosis results are better than side to side anastomosis.

## Introduction

 Arteriovenous fistula is the treatment of choice for hemodialysis in chronic renal failure patients. Back in early 20^th^ century the arteriovenous shunting was started for hemodialysis.^1^ In 1950’s, the standard Quinton-Scribner silastic Teflon shunt infection and thrombosis were the main complications of arteriovenous shunts. Spontaneous dislocation was also a major issue.^1^ To overcome this problem Brescia, Cimino, and Hurlwith made surgically created fistulae between cephalic vein and radial artery at the wrist. Such type of vascular access for hemodialysis was first described in the 1966.^2^ Currently it is the mainstay of treatment of chronic renal failure patients. The data from U.S, interpret that prevalence of arteriovenous fistulae increased from 33% in 2003 to 41% in 2005 while the incidence increased from 26.3% in 1999 to 36.3% in 2003.^3^

 Chronic renal failure patients had enormous benefits from these fistulae, as these have better safety profile and long-term patency.^4^^,^^5^ Chronic renal failure patient may have unsuitable veins for native arteriovenous fistula.^6^ Severe arteriosclerosis, edema of the arm, vascular calcification, multiple vein puncture are usual problem.^7^ To overcome these difficulties other fistula sites in different parts of body have been recommended such as, antecubital area of non-dominant arm where Radial or Brachial artery could be anastomosed with Cephalic or Basalic vein.^5^^,^^8^^,^^9^

 However selection of brachial artery could pose risk of significant cardiovascular complication and due to high brachial artery bifurcation (HiBAB). Moreover this site provides limited space for venipuncture for hemodialysis purpose.^10^^,^^11^

## Methodology

 This prospectively designed case series study was conducted at Department of Urology, JPMC, Karachi, from 1^st^ January 2009 to 31^st^ December 2010. Patients were chosen for CBRC arteriovenous fistula at wrist after the evaluation of normal radial pulse and distensible veins at the wrist of non-dominant arm. Patients undergoing other types of vascular access or secondary fistula formation were excluded. Pre-operative evaluation included detailed evaluation of vessels clinically, quality of artery and vein, pin pointing the main arterial supply of the hand by Ellens test. Status of the arm whether dominant or non-dominant (some patients could be having left dominant arm), functional or paralyzed arm, previous vein puncture or edema or trauma were assessed.

**Table-I:** Descriptive statistics

		*Frequency*	*Percentage*
Gender	Male	102	56%
	Female	80	44%
Side of AVF	Left	173	95%
	Right	09	5%
Site of AVF	Radio cephalic	114	62.6%
	Radial artery with Median antebrachial vein	10	5.5%
	Radial artery with Venacumitant	02	1.1%
	Brachial artery with Venacumitant	01	0.5%
	Ante-cubital	55	30.2%
	Fistulae failure 11 month	23	12.6%
	Patent fistulae at 3 months	121	66.5%
	Patent fistulae at 6 months	105	57.7%

 Ipsilateral presence of subclavian double lumen catheter or its scar marks which might pose a risk of engorged superficial shoulder vein further confirmed the diagnosis. All the fistula formation ware performed under local anesthesia. Transverse incision was made in antecubital fossa to expose Brachial artery, Basalic and Cephalic vein and vertical incision was made at forearm for Radial artery and Cephalic vein. Anastomosis was created in an end to side or side to side fashion depending upon mobility of target vein. The distal vein was ligated in case of radio-cephalic anastomosis. Heparin was not administered routinely during or after fistula formation. Prolene 6/0 suture was always used. Haemostasis was checked and secured. Skin was closed with prolene 3/0. Non-circular dressing was applied and postoperative hand exercises were advised. 

**Table-II:** Causes of chronic renal failure

*Causes of CRF*	*Frequency*	*Percentage*
Diabetes Mellitus	77	42%
Hypertension	59	32%
Both DM & hypertension	19	10%
Chronic glomerulonephritis	08	4.4%
Polycystic kidney disease	03	1.6%
Renal atrophy	01	0.55%
Minimal change glomerulonephritis	01	0.55%
Secondary primary postpartum haemorrhage	02	1.1%
Rapidly progressive glomerulonephritis (RPGN)	02	1.1%
Obstructive nephropathy	07	3.8%
Chronic interstitial nephritis	03	1.6%

 All operations were performed by the researcher and supervised by the senior consultant. AVF maturation time proposed was one month. Patients were followed for 03 to 06 months. Primary failure occurred if the fistula never matured adequately, dialysis was no longer possible via this access site or intervention was required to maintain fistula function. 

## Results

 One hundred and eighty two patients underwent arteriovenous fistula formation. The mean ± SD age of patients was 63 ± 13 years and there were 102 (56%) males and 80 (44%) females. 173 (95%) had left non-dominant arm and 09 (5%) right non-dominant arm. Radiocephalic arteriovenous fistulae were 114, 10 had Radial artery with Median antebrachial vein, 02 had Radial artery with venacomitant and 55 were Antecubital arteriovenous fistula made by anastomosis between Brachiocephalic, Basalic, Median cubital and 1 anastomosis were brachial artery with venacomitant (Table-I).

 Medical history included diabetes in 77 (42%), hypertension in 59 (32%) patients and 19 (10%) had both history of hypertension and diabetes mellitus. Other associated diseases were found in 27 (15%). These were chronic glomerulonephritis 08 (4.4%), polycystic disease 03 (1.6%), renal atrophy 01 (0.55%), Minimal change glomerulonephritis 01 (0.55%), secondary primary postpartum haemorrhage 02 (1.1%), rapidly progressive glomerulonephritis 02 (1.1%), obstructive nephropathy 07 (3.8%) and chronic interstitial nephritis 03 (1.6%) (Table-II).

 Twenty three (12.6%) fistulae failed within the first month without dialysis. The primary patency rate was 121 (66.5%) at 3 months and 105 (57.7%) at 6 months (Table-I). Failing arteriovenous fistulae were managed by new arteriovenous fistula in our series, 52 (28.6%) patients had redo arteriovenous fistula made.

 The study also demonstrated a poor outcome for fistulas in diabetic patients; as 15 out of 23 (65.2%) who failed primarily were diabetics and out of these 13 (86.7%) failed in first 3 months.Analysis of complications, infection and burst fistulae was found in 9 (4.91%), pseudo aneurysm in 06 (3.27%), fever 09 (4.91%), peri-operative failure 01 (0.55%) and burst fistulae 06 (3.27%) (Table-III).

## Discussion

 In this study 182 patients underwent CBRC arteriovenous fistula at wrist and were followed-up for two years. Results showed that first month failure rate in these fistulae were 12.6% even without dialysis while primary patency rate was 66.5% at three months and 57.7% at six months.

 Arteriovenous fistula is a difficult procedure for urologists because it involves vascular surgery as well as for patients because it is painful and cumbersome, yet the urologists have to take up the task for chronic renal failure patients.^12^ Preoperative evaluation is very important to avoid any operative or postoperative catastrophe.^13^ The radiological mapping should be reserved for difficult cases.^14^^,^^15^ Radiocephalic fistula is performed more successfully due to the adequate length of arteries and veins.^5^ We think meticulous clinical evaluation is sufficient.

 Kakkos SK, et al;^16^ documented patency rates of 70% at 12 months and 58% at 18 months and compared these with transposed brachial-basilic fistulas in which patency rates were 82% at 12 months and 78% at 18 months. In our study failure in 12.5% of fistulae may be due to failure of the vein to mature adequately or thrombosis. Fistula failure after six months might have been associated with vein stenosis followed by thrombosis. Heparin was not used in our patients. Close hemodynamic monitoring during dialysis has been advocated, by measuring venous pressure, arterial inflow and recirculation, to detect stenosis.^17^ In another study 24 months post clot removal patency was found to be 50-86%.^14^

**Table-III:** Complications of arteriovenous fistula

*Complications*	*Frequency*	*Percentage*
Infection + Burst fistulae	9	4.91%
Pseudo aneurysm	6	3.2%
Fever	9	4.91%
Peri-operative failure due to Bleeding	01	0.55%
Burst fistulae	06	3.2%

 Patency of arteriovenous fistula may be influenced by patient factors, techniqueand core of the fistula following operation. Ideally, in assessing the influence of patient related variables on fistula patency, other factors should be kept constant. Rapid High-flux hemodialysis might lead to less favorable fistula patency.^18^ Rapid fluid shifts may predispose to thrombosis while the higher flow rates (mean flow rate in functioning fistulas was around 400ml/min) may uncover any fistula abnormalities earlier. Using this practice the patency rate in our study at three months was 66.5% and at six months was slightly lower than with those reported for conventional dialysis.

 Kumar A, et al;^19^ demonstrated highly variable out comes for radio cephalic fistulas compared to brachiocephalic fistulae, the incidence of vascular steal syndrome was extremely rare (0%) in radiocephalic as compared to much higher (20%) in brachiocephalic fistulae. The patency rate at 24 months varied between 13 and 62 percent for different operators. Fassiadis N, et al;^20^ described that there is effect of surgeon’s hand in increased patency or fistulae. The relatively poor prognosis of fistulas created in women is supported by other studies. In an older study, Wong V, et al; described that there was poor outcome of fistulas in women, which may be partly explained by their smaller vessels.^21^ The recent evidence from national and international studies deny any affect of sex on duration of patency of arteriovenous fistulae.^22^^,^^23^ They further found that all vessels used for fistulas with diameters of 16mm or less invariably failed within 12 weeks. Patients with post-operative fistula failure due to infection or hematoma were not destined to salvage procedure as international studies reveal disappointing results, therefore we preferred fistula ligation in these patients followed by reestablishment of new arteriovenous fistula at suitable other site.

 End to side arteriovenous fistula proved to have better results than side to side arteriovenous fistula in our series. It could be due to tension free anastomosis with longer patency rate. International literature series recommend the use of magnifying loupe over naked eye technique, which was used in all our cases with equally good outcomes.^24^^,^^25^

 In this study diabetic patients of either sex had a poor outcome for these fistulae as from 77 (42%) diabetics in the study, 65.2% failed primarily and 86.7% failed in first three months. Leapmen and colleagues^26^ also demonstrated the poor out come for diabetic patients and reported even lower patency rates of 42% at 12 months and 18% at 60 months. Similarly, Monroy-Cuadros M and colleagues^27^ found diabetes to be a significant risk factor for failure of fistulas within the 1st year. Diabetic patients also have a poor out comes with prosthetic fistula. The poorer results in diabetics might be explained by increased arterial disease or even impaired venous endothelial function.^28^ It could also be due to infectious complication because of immuno-suppression in addition sclerosed arteries also make anastomotic techniques difficult.

 In a recent meta-analysis, Lazarides and colleagues^29^ found age to be significantly associated with failure of arteriovenous shunting and suggested proximal autologous brachiocephalic fistulas in elderly patients which was in earlier studies^30^ was found to produce adverse outcomes. Tordoir et al^31^ found different results, where in a much smaller series of fistulas followed closely by duplex surveillance, they found age to be a non-significant factor. These results were replicated in our study and there was no evidence that older patients have a poorer prognosis.

 We have made many fistulas utilizing median antebrachial vein in 10 patients, venacomitant in 03 patients. More arteriovenous fistulae were not possible at other standard sites due to other reasons. It was observed that these veins have thin wall and are difficult to arterialize. Some patients had patent veins for three months.

## Conclusions

 About one-third of radiocephalic fistulas fail within two years. The outcome is worse for women and diabetic patients. This information may be useful in assessing and counseling patients with end-stage renal failure. Arteriovenous fistula is the better and ideal choice for hemodialysis. Radiocephalic fistulae in forearm seems to have better results in comparison to cubital fossa arteriovenous fistula. End to side anastomosis gives better results than side to side anastomosis.
